# Phases of methodological research in biostatistics—Building the evidence base for new methods

**DOI:** 10.1002/bimj.202200222

**Published:** 2023-02-03

**Authors:** Georg Heinze, Anne-Laure Boulesteix, Michael Kammer, Tim P. Morris, Ian R. White

**Affiliations:** 1 Center for Medical Data Science, Institute of Clinical Biometrics, Medical University of Vienna, Vienna, Austria; 2 Institute for Medical Information Processing, Biometry and Epidemiology, Ludwig-Maximilians University of Munich, Munich, Germany; 3 Department of Medicine III, Division of Nephrology, Medical University of Vienna, Vienna, Austria; 4 MRC Clinical Trials Unit, Institute of Clinical Trials & Methodology, University College London, London, UK

**Keywords:** biostatistics, methodological research, reproducibility

## Abstract

Although new biostatistical methods are published at a very high rate, many of these developments are not trustworthy enough to be adopted by the scientific community. We propose a framework to think about how a piece of methodological work contributes to the evidence base for a method. Similar to the well-known phases of clinical research in drug development, we propose to define four phases of methodological research. These four phases cover (I) proposing a new methodological idea while providing, for example, logical reasoning or proofs, (II) providing empirical evidence, first in a narrow target setting, then (III) in an extended range of settings and for various outcomes, accompanied by appropriate application examples, and (IV) investigations that establish a method as sufficiently well-understood to know when it is preferred over others and when it is not; that is, its pitfalls. We suggest basic definitions of the four phases to provoke thought and discussion rather than devising an unambiguous classification of studies into phases. Too many methodological developments finish before phase III/IV, but we give two examples with references. Our concept rebalances the emphasis to studies in phases III and IV, that is, carefully planned method comparison studies and studies that explore the empirical properties of existing methods in a wider range of problems.

## 1 Introduction

Career plans and funding calls in biostatistical methodology often revolve around “novelty” and “innovation.” This stimulates the development of new methods, and leads to the publication of results that show a new method working well. For example, asymptotic properties of a method are established and the finite-sample case investigated using simulation studies. Recently, it was demonstrated that how “new” methods can easily be proven to be optimal using simulation studies (Pawel et al., 2022). A paper introducing a new method and demonstrating its superiority over existing methods with simulation studies should therefore be treated with caution; we must be aware that these simulations may be prone to inventor bias (Boulesteix et al., 2013). Narrow asymptotic results and simulation studies may not create a sufficiently broad base of evidence to ensure the trustworthiness of that method. While new methods are essential to solve existing and new problems, users of methods need to understand which methods work well when. A trustworthy method keeps its essential operational characteristics in a wide variety of settings where it might be applied, or is sufficiently well understood such that a user of the method would know when to use the method and when to avoid it. More and more new methods are proposed without ever being fully investigated and adequately compared in a wider variety of situations. This creates the problem that, even though there is a plethora of methods available to the analyst, many of them are not trustworthy enough to be used in practical analyses. In order to improve this unfortunate situation, we propose a framework to think about how a piece of methodological research contributes to the evidence base for a specific method. A much needed side-effect is that such a concept gives more gravitas to carefully planned method comparison studies and to studies that explore the empirical properties of existing methods in a wider range of problems (Boulesteix et al., 2018). All authors of this paper are members of the international stratos Initiative (STRengthening Analytical Thinking for Observational Studies) and support the initiative’s overarching aim to provide guidance for relevant methodological topics in the design and analysis of observational studies for specialist and non-specialist audiences (Sauerbrei et al., 2014). The proposed framework aims at refining the notion of evidence in methodological research that is central to STRATOS’ efforts.

## 2 Learning From Drug Development

In drug development, the concept of *phases of research* was defined decades ago (Sedgwick, 2014). As research progresses from one phase to the next, many candidate treatments are dismissed because of intolerability (phase I), lack of safety or of efficacy (phase II), or ineffectiveness when compared to a placebo or standard of care (phase III), while promising treatments advance to the next phase. After licensing of a drug, a phase IV trial investigates long-term effects and effectiveness in the real world; this may allow identification of, for example, an expanded safety profile, expansion of indication or treatment effect heterogeneity. Previous work has also defined *phases of prognostic factor research* to identify underlying methodological issues and provide guidelines for the conduct of prognostic factor studies (Altman & Lyman, 1998; Hayden et al., 2008; Riley et al., 2013). Here, also four phases were defined, where in phase I exploratory, hypothesis generating studies would propose a new prognostic marker to have prognostic importance, and in phase II exploratory studies would attempt to use the marker to discriminate between patients at high and low risk of disease progression (prognostic ability) or to identify which patients are likely to benefit from therapy (predictive ability). Phase III would be confirmative studies to proof a priori hypotheses about the prognostic or predictive abilities of the marker. Finally, further studies may combine several prognostic markers into a prognostic or predictive model.

We argue that a similar concept of “phases with well-defined aims” helps to build the evidence base for methodological research.

The aim of methodological research is to give applied researchers methods to obtain accurate answers to relevant questions (and to identify methods that fail to do this), along with the necessary understanding to use the methods properly. Similarly, the aim of drug development is to precisely estimate a drug’s beneficial and adverse causal effects in various potential application areas. In drug development, regulatory involvement ensures that the development phases achieve these aims, being efficient with early pulling out for unpromising drugs. In methodological research, just as in drug development, new methods can be worse than existing ones or have unexpected properties in some situations. For promising methods, it is not just a matter of introducing the method and getting it used; similarly to a drug, it needs to be carefully evaluated broadly in a way that onlookers can trust. Further, just as in drug development, existing methods may be repurposed. While we do not envision a single governing body that regulates this, methodological research may benefit from considering how evidence is created through the phases in drug development.

## 3 Introducing A Framework Of Phases Of Methodological Research

Methodological phase I may introduce a new idea and try to prove that the proposed method is valid from a theoretical point of view and has the potential to improve on existing methods, or may constitute the first solution to a particular problem. It often includes logical reasoning, proofs and investigation of asymptotic properties such as consistency or normality, etc. This does not mean that further proofs and investigations of asymptotic properties in a wider variety of situations will not be needed in later phases. In practice, phase I studies’ results may often reveal no or only a small benefit of a new method and researchers do not try to disseminate them or, if they do, some journals may be reluctant to publish them. We claim that such “negative” studies, if they can explain why a method does not work in a specific situation, are sometimes needed to increase the community’s understanding, to stimulate further research and to stop other researchers from investing time and resources in the same dead-end idea (Boulesteix et al., 2015).

Methodological phase II may have the aim to prove that a method can be used with caution in an applied setting which is not completely identical to the developer’s target setting. A phase II study may provide empirical evidence to demonstrate validity in finite samples using simulation studies with a limited set of scenarios, or by illustrative data analyses. When browsing the table-of-contents of typical biostatistical journals, one gets the impression that phase II study reports are abundant in the biostatistical literature (see also below). Often, a given paper includes both phases I and II contributions. By the end of phase II, an openly available software implementation of the method may facilitate the uptake of the method for “early adopters” and ease further investigation in later phases.

Methodological phase III may investigate how the method performs in a wide range of settings and, if applicable, for different types of outcomes. This may include empirical comparisons with any existing methods. From such studies, researchers may learn in which situations and under which assumptions the method can be safely used and performs better than or at least as well as alternative methods. This includes understanding which of the method’s assumptions are critical and which are not: for example, in linear regression with large samples, the assumption of normally distributed residuals is not critical in terms of consistency of point and variance estimators. Hence, a phase III study must provide substantial evidence to demonstrate a method’s validity and relative performance. It should be replicable (Lohmann et al., 2022) and, if possible, avoid “inventor bias” by making efforts to ensure neutral comparisons (Boulesteix et al., 2013) or at least disclose possible biases. Furthermore, it typically includes “broad” simulations in different, practically relevant settings. Phase III may detect previously unknown implicit assumptions of a method. Methods that were not properly validated may show undesirable or unintended properties when applied in a situation, where such an assumption is not met. Several examples involving real data would have to demonstrate how to properly apply the method in question and how to interpret its results. By the end of phase III, a software implementation of the method should be reasonably fast and user-friendly to be applicable for a wider audience.

Methodological phase IV should establish that a method is now suitably well-understood, that is, we know when it is the preferred method and when it is not. A phase IV study is based on extensive experience with the method. For example, a phase IV study may systematically review applications of a method, or may deal with applying the method in new settings that were not considered initially. In this phase, pitfalls of a method may be discovered and highlighted, that is, things that are likely to go wrong if the method is applied carelessly by a user. Likewise, a phase IV study may propose essential, practically useful diagnostics that help a data analyst to assess if any critical assumptions of a method were violated for the data in hand. Using simulations, new mathematical results (Wang et al., 2019) and example analyses of interesting case studies beyond previous applications of the method, it may identify “sweet spots” and “breakdown scenarios” for a method (by analogy to optimal use and long-term or rare adverse effects of a drug). Breakdown scenarios in which the method gives suboptimal results may not have been obvious when the method was introduced and may only be discovered through extensive experience, for example, also by evaluating its behavior when competing methods are known to break. Such breakdown scenarios may give rise to modifications and further developments. A modification may make the method applicable in further settings, for which it may undergo another phase II and possibly phase III. After that phase III evaluation, it may turn out that the modification is suitable only for very special target settings. In some areas, such as machine learning, “adversarial examples,” that is, analysis situations or datasets where a method fails, are frequently published, and often stimulate research toward robustifying existing methods (Biggio & Roli, 2018). In biostatistics, this is very rarely the case, or such examples are hidden in phase II studies intended to motivate the need for another method. Nevertheless, empirical studies on the breakdown of a method, particularly if they contain explanations of why a breakdown happens, will increase our understanding, prevent others from wasting their time on it, and are therefore worth publishing. By phase IV, a robust implementation of the method should be easily accessible to practitioners and the understanding of the method so advanced that in principle alternative implementations could be developed in several software packages.

The different phases of research may be summarized by different scopes, elements, and outcomes of a study (Table 1). For example, studies in phase I will often focus on the introduction of a new method, while only later comparisons become more important and lastly, in phase IV the focus is more on investigating where a method works and where it fails in a broad spectrum of applications.

## 4 Examples

For a given method, it is still unusual to have all four phases of methodological research represented in publications. As positive exceptions, we describe two developments representing some of the authors’ interests.

### 4.1 Example 1: Firth’s correction

Firth’s correction is a bias correction method for maximum likelihood estimators. As a side effect, the correction gives finite estimates of regression coefficients in generalized linear models even with data constellations, where maximum likelihood estimates do not exist.

Phase I: In his 1993 paper in Biometrika, David Firth presented the correction for the first time, derived it algebraically, and gave some simple examples (Firth, 1993).

Phase II: In 2002, Heinze and Schemper took up the idea and provided, for the first time, evidence from a simulation study with logistic regression with binary covariates, demonstrating that the method improved on previously available methods to deal with non-existing maximum likelihood estimates (Heinze & Schemper, 2002).

Phase III: A comprehensive simulation study on logistic regression was performed by van Smeden and colleagues in 2016 using 465 scenarios (van Smeden et al., 2016). The study confirmed the earlier results that regression coefficients are less biased and more precise when estimated using Firth’s correction. The study was intended as neutral in that none of the authors had published work on Firth’s correction. Similar results were obtained by Puhr et al., who also suggested two modifications of Firth’s correction, both of which make probabilities predicted from the model more precise compared to using the original correction, while retaining the favorable properties of the estimators of the regression coefficients (Puhr et al., 2017). These modifications were compared to some Bayesian approaches using weakly informative priors which—in the meantime—had been suggested as alternative solutions to solve the problem.

Phase IV: In 2018, Mansournia and colleagues summarized the evidence on the topic and explained failure of maximum likelihood estimation and solutions by means of two data examples (Mansournia et al., 2018). They also explained why different methods to deal with separation lead to different results and gave general advice on how to detect and to deal with separation in practice.

### 4.2 Example 2: Predictive mean matching

The literature on multiple imputation is vast and includes several strands that have effectively undergone phases of development. Here, we review predictive mean matching, a type of “hot deck” procedure that multiply imputes each missing value with a “borrowed” observed value.

Phase I: The idea of predictive mean matching was introduced by Little (1988) as a way of imputing only observable values by replacing missing values with observed values of “donors,” based on a model. There was no proof of its validity but the idea was linked to multiple imputation and Rubin’s theoretical work on hot deck multiple imputation procedures, published a year earlier (Rubin, 1987). A multivariate imputation extension was outlined.

Phase II: Heitjan and Little (1991) used predictive mean matching to multiply impute seatbelt use and blood alcohol content in the Fatal Accident Reporting System (fars) database. This was followed by a limited simulation study, with data generation involving the fars data, to evaluate the performance of predictive mean matching. The simulation results showed promising, though not ideal, performance.

Phase III: Schenker and Taylor (1996) conducted a simulation study which, in particular, compared methods for identifying “donors” when using predictive mean matching. They conducted a reasonably broad simulation study with incomplete continuous outcomes. This explored the performance of three predictive mean matching variants, among other methods, and found that they performed reasonably well across a range of scenarios.

Phase IV: Morris et al. (2014) reviewed the existing literature on predictive mean matching and considered how to “tune” its implementation. Some of their simulation studies used scenarios where predictive mean matching might be expected to break: for example, with small-sample size and data strongly missing at random (i.e., with missingness depending strongly on observed variables). This showed when the method performed poorly, and how poorly, and clarified how and when it would be expected to outperform alternative methods.

### 4.3 Classifying articles into phases: A pilot study

In a pilot evaluation of the phases that are published, we analyzed a volume of each of four biostatistical journals. The evaluation revealed that most articles of an issue of *Biometrika* dealt with phase I studies, while phase II dominated in *Biometrical Journal, Statistics in Medicine* and *Statistical Methods in Medical Research*. Overall, only a few papers were found that could be classified as phase IV. The protocol and detailed results of the pilot study can be found in the Supporting information. This pilot study had several limitations. First, it was based on the judgment of the main phase contribution and many papers will span more than one phase. Second, papers were assessed by a single evaluator only and the judgment may vary between different evaluators. Third, for simplicity the pilot study was conducted with a one-rater-one-journal design, so a journal’s assessment was probably confounded with personal judgment.

## 5 Further Steps

Our proposal aims to provide a framework to communicate about the development stage of a method, that is, to understand the limitations of current research, and what further work would be necessary for wide understanding and application of a method. So far we have sketched how the phases of methodological research could be defined, but one may also think of phases when developing software packages to implement methods. In order to define phases such that they are useful and practical for the scientific community, a larger systematic assessment of methodological papers in different biostatistical journals building on our pilot study, and a Delphi process aiming at reaching an agreement on the definitions, are probably needed. After such work, a tool could be developed that enables a methodologist to assess and specify the phase of their research. The technology readiness level calculator of NASA may be template for such a tool (Altunok & Cakmak, 2010). In addition, signaling questions may help to rule in or rule out certain phases. Given a broad consensus and existence of guidance for phase identification, the wide adoption of such a framework may facilitate efficient communication and “peer-regulation” of the current state of knowledge about a method. Thereby, the framework would increase trustworthiness in the methodological development process, which is to the benefit of scientific communication, that is, it helps authors, journal editors, and readers and reviewers of manuscripts and grants. The ultimate goal is to ensure that users of methods are equipped with a solid evidence base that allows them to choose the appropriate method for a given challenge. Transparently labeled methodological studies of whatever phase may also stimulate other biostatistical researchers to get interested in a method or a methodological problem and may encourage them to conduct a study in the next phase. For example, good phase IV studies which show shortcomings of existing methods can help focus thought on solutions, and so may lead to “inventing” better methods.

Research in all four phases is important for scientific advancement, but currently there are many obstacles to achieving an appropriate balance. First, many funding agencies are inclined to fund only early phase methods research while unrealistically expecting “phase-IV-like results” within a too short time frame, and many biostatistical journals favor papers on new methods over articles comparing existing methods. Similarly, early career methodologists are often pushed to publish “original” research in order to get tenure. However, the classical definition of *originality* is a very narrow one. If this same standard were applied to funding for randomized trials, we would not have studies like Recovery (Nuffield Department of Publication Health, 2022) or Stampede (MRC Clinical Trials Unit at UCL, 2022) which re-examine existing treatments in new and highly relevant settings. We claim that phases III and IV methodological studies are undervalued in the statistical community and often downplayed as “yet another” simulation study or application, yet planning and conducting properly designed studies covering a broad variety of clearly defined settings is not trivial. Successful phase III or phase IV research involves careful consideration of the practical impact of the methods: selection and implementation of reasonable simulation setups, identification of relevant example datasets, finding out how the methods might be used in practice and extracting relevant evidence from the numerical results, as well as a good working understanding of the methods themselves. Such studies provide novelty by increasing the scientific evidence base for methods and extending the scope of their safe applicability and hence are as important to scientific advancement as “inventing” a new statistical method. The acceptance of phase III and IV studies could be increased by introducing new ideas related to the design and conduct of simulation and comparison studies toward the same rigor as clinical trials. This includes, for example, clarifying the assumptions and range of problems that the study seeks to address, publishing a protocol before conducting the study (Kipruto & Sauerbrei, 2022), or distributing the roles of data generator, data analyst, and performance evaluator between different parties. Furthermore, inventor bias should be identified and avoided or at least disclosed (Couronné et al., 2018; Herrmann et al., 2021).

Concerning the reporting of such studies, the main task is to transparently clarify the level of trustworthiness of methods, both in absolute and comparative terms. The biostatistical community would benefit from phase IV studies, especially when these studies clarify when a method can—rather than cannot—be recommended for the task at hand. Experienced applied statisticians may to some extent develop a good gut feeling for this difficult task, but phase IV studies would provide the objective evidence for this intuition and aid decision making for less experienced researchers.

While some of our ideas may seem ambitious and not easy to reach within short term, we are confident that a discussion on phases of methodological research, inspired by this paper, will reach short-term goals such as:

(i) Giving frustrated researchers doing early-phase work a framework to understand why their method has not been universally adopted, and suggestions on how to achieve wider adoption: for example, by conducting broad comparison studies while acknowledging possible biases, by supporting neutral comparisons and by making methods easily accessible to other researchers.
(ii) Giving applied researchers a way to articulate their scepticism about new methods that have not undergone several phases of testing.
(iii) Giving more legitimacy to phase III and IV studies and encouraging researchers to conduct and journal editors to publish them.

## 6 Conclusion

We believe that a framework such as the one outlined in this paper may make method development more trustworthy, provide an efficient tool to communicate about methods’ applicability, and increase visibility of research concerned with making the applications of methods safe and successful. Rather than the vague, cliched “more research is needed,” our framework provides a constructive way of thinking about *what* research would move the development of a method forward.

## Supplementary Material

Supporting Information

## Figures and Tables

**Table 1:** A brief description of the proposed scheme of phases of methodological research

Phase	Scope: A study in that phase will typically aim at…	Elements: Typically, a study in that phase will consist of…	Outcome: after that phase, we know…
I	… introducing a new idea, demonstrating its validity by investigation of (asymptotic or finite-sample) properties, showing potential to improve on existing methods or to be the only solution.	… mathematical derivations and proofs, very simple example data analyses.	… whether a method is valid or invalid from a theoretical point of view.
II	… demonstrating the use of the method with real data, probably introducing refinements and extensions; it will consider only a limited range of possible applications.	… simulations including limited comparisons with other methods, simple example data analyses.	… whether a method can be used with caution or should not be used in certain applied settings.
III	… comparing a relatively new method with competitors and demonstrating its use in practice; it will consider a wide range of applications.	… simulations with wide range of scenarios and different outcome types (ideally set up as neutral comparison studies), realistic comparative example data analyses.	… in which settings (among many) a method can be safely used and in which it outperforms competing methods.
IV	… summarizing the evidence about a method, also in comparison with competing methods; uncovering previously unknown behavior of the method in complex data analyses; considering an extended range of possible and actual applications.	… a review of the existing evidence about a method, simulations with extended range of scenarios, complex comparative example data analyses.	… when a method is and when it is not the preferred method; what diagnostics are available and which pitfalls may occur with its application.

## Data Availability

The full data of the pilot study can be found in the Supporting information.
