# Supplementary material for: Phases of methodological research in biostatistics—Building the evidence base for new methods
Source: Biom J. Author manuscript; Available in PMC 2024 Jan 11. (PMC7615508; doi:10.1002/bimj.202200222)
Supplement: Supporting Information [file EMS177446-supplement-Supporting_Information.docx]

Phases of methodological research in biostatistics – building the evidence base for new methods

Georg Heinze, Anne-Laure Boulesteix, Michael Kammer, Tim P. Morris, Ian R. White on behalf of the Simulation Panel of the STRATOS initiative

Supplementary material: Representation of phases of methodological research in the biostatistical literature – a pilot study

# Aim

The aim of this pilot study was to get a first idea of the prevalences of different phases of methodological research in the biostatistical literature.

# Method

The evaluation was based on a preliminary definition of the phases of methodological research as proposed by I.W. and agreed on in June 2021 between all authors:

| - Phase I: Method is valid and has the potential to improve on existing methods. - Phase II: Method can be used with caution in an applied setting. - Phase III: Method is understood well enough that it can be safely used in a range of settings. This includes knowing how to check its assumptions and understanding which assumptions are critical and which are not. - Phase IV: Method is understood well enough that it is known when it is and when it is not the preferred method. |
| --- |

The following four biostatistical journals were chosen: Biometrical Journal, Statistics in Medicine, Statistical Methods in Medical Research, Biometrika.

Four authors each chose a journal, selected a recent issue and assigned each article of that issue to one of the four phases. The chosen issues were:

| \| Journal \| Issue \| Number of articles \| Assessed by \| \| --- \| --- \| --- \| --- \| \| Biometrical Journal \| Volume 63, Issue 4 (April 2021) \| 12 \| G.H. \| \| Statistics in Medicine \| Volume 40, Issue 15 (July 2021) \| 12 \| A.L.B. \| \| Statistical Methods in Medical Research \| Volume 30, Issue 5 (May 2021) \| 13 \| M.K. \| \| Biometrika \| Volume 108, Issue 2 (June 2021) \| 16 \| T.M. \| |
| --- | --- | --- | --- | --- | --- | --- | --- | --- | --- | --- | --- | --- | --- | --- | --- | --- | --- | --- | --- | --- |

# Results

The following table summarizes the results of the pilot evaluation:

| Phase | Biometrical Journal | Statistics in Medicine | Statistical Methods in Medical Research | Biometrika |
| --- | --- | --- | --- | --- |
| 0 (pre I) |  |  |  | 3* |
| I | 0 | 2 |  | 9** |
| I or II | 2 |  | 3 |  |
| II | 7 | 8 | 7 | 4 |
| II or III |  |  | 1 |  |
| III | 0 | 2 | 2 |  |
| IV | 1 |  |  | 1** |
| Not classifiable | 1 |  |  |  |

* 3 papers were rated as rather theoretical with no application in mind.

** 1 paper was rated as mix of phases I and IV.

# Details of the evaluation

## Biometrical Journal Volume 63(4)

1. Meta-analysis of diagnostic accuracy studies with multiple thresholds: Comparison of different approaches

https://doi.org/10.1002/bimj.202000091

Phase IV

2. Weighted estimators of the complier average causal effect on restricted mean survival time with observed instrument–outcome confounders

https://doi.org/10.1002/bimj.201900284

Phase II

3. Joint frailty model for recurrent events and death in presence of cure fraction: Application to breast cancer data

https://doi.org/10.1002/bimj.201900113

Phase II

4. Statistical method for modeling sequencing data from different technologies in longitudinal studies with application to Huntington disease

https://doi.org/10.1002/bimj.201900235

Phase ? (not classifiable)

5. Analyzing longitudinal clustered count data with zero inflation: Marginal modeling using the Conway–Maxwell–Poisson distribution

https://doi.org/10.1002/bimj.202000061

Phase II

6. Nonparametric Bayesian functional two-part random effects model for longitudinal semicontinuous data analysis

https://doi.org/10.1002/bimj.201900280

Phase I/II

7. Power and sample size for random coefficient regression models in randomized experiments with monotone missing data

https://doi.org/10.1002/bimj.202000184

Phase II

8. Statistical inferences on nonconstant relative potency with quantal response data

https://doi.org/10.1002/bimj.202000073

Phase II

9. A parametric quantile regression approach for modelling zero-or-one inflated double bounded data

https://doi.org/10.1002/bimj.202000126

Phase II

10.Generalised M-quantile random-effects model for discrete response: An application to the number of visits to physicians

https://doi.org/10.1002/bimj.202000180

Phase II

11.Ecological hazard assessment via species sensitivity distributions: The non-exchangeability issue

https://doi.org/10.1002/bimj.201900404

Phase I/II

12.Correcting the bias of the net benefit estimator due to right-censored observations

https://doi.org/10.1002/bimj.202000001

Phase II/III

## Statistics in Medicine Volume 40(16)

1. Selection of within-run quality control rules for laboratory biomarkers

https://onlinelibrary-wiley-com.emedien.ub.uni-muenchen.de/doi/10.1002/sim.8987

Phase II-III

2. Computing the polytomous discrimination index

https://onlinelibrary-wiley-com.emedien.ub.uni-muenchen.de/doi/10.1002/sim.8991

Phase II

3. Cost-efficient clinical studies with continuous time survival outcomes

https://onlinelibrary-wiley-com.emedien.ub.uni-muenchen.de/doi/10.1002/sim.8992

Phase II

4. Confidence interval estimation for sensitivity and difference between two sensitivities at a given specificity under tree ordering

https://onlinelibrary-wiley-com.emedien.ub.uni-muenchen.de/doi/10.1002/sim.8993

Phase II

5. Regression analysis of arbitrarily censored survival data under the proportional odds model

https://onlinelibrary-wiley-com.emedien.ub.uni-muenchen.de/doi/10.1002/sim.8994

Phase II

6. Measuring association among censored antibody titer data

https://onlinelibrary-wiley-com.emedien.ub.uni-muenchen.de/doi/10.1002/sim.8995

Phase II

7. Bayesian semiparametric mixed effects models for meta-analysis of the literature data : An application to cadmium toxicity studies

https://onlinelibrary-wiley-com.emedien.ub.uni-muenchen.de/doi/10.1002/sim.8996

Phase I

8. Using generalized linear models to implement g-estimation for survival data with time-varying confounding

https://onlinelibrary-wiley-com.emedien.ub.uni-muenchen.de/doi/10.1002/sim.8997

Phase I

9. A multistate survival model of the natural history of cancer using data from screened and unscreened population

https://onlinelibrary-wiley-com.emedien.ub.uni-muenchen.de/doi/10.1002/sim.8998

Phase II

10. Score tests for scale effects, with application to genomic analysis

https://onlinelibrary-wiley-com.emedien.ub.uni-muenchen.de/doi/10.1002/sim.9000

Phase II

11. Semiparametric recurrent event vs time-to-first-event analyses in randomized trials: Estimands and model misspecification

https://onlinelibrary-wiley-com.emedien.ub.uni-muenchen.de/doi/10.1002/sim.9002

Phase III

12. Nowcasting COVID-19 incidence indicators during the Italian first outbreak

https://onlinelibrary-wiley-com.emedien.ub.uni-muenchen.de/doi/10.1002/sim.9004

Phase II

## Biometrika Volume 108(2)

1. A general interactive framework for false discovery rate control under structural constraints

https://academic.oup.com/biomet/article/108/2/253/5879274

Phase I

2. Approximating posteriors with high-dimensional nuisance parameters via integrated rotated Gaussian approximation

https://academic.oup.com/biomet/article/108/2/269/5897408

Phase II

3. Statistical properties of sketching algorithms

https://academic.oup.com/biomet/article/108/2/283/5878938

Phase I with a bit of phase IV(!)

4. Quasi-oracle estimation of heterogeneous treatment effects

https://academic.oup.com/biomet/article/108/2/299/5911092

Phase I

5. Inference for treatment effect parameters in potentially misspecified high-dimensional models

https://academic.oup.com/biomet/article/108/2/321/5902826

Phase I

6. Specification tests for covariance structures in high-dimensional statistical models

https://academic.oup.com/biomet/article/108/2/335/5903732

Phase I

7. On the use of a penalized quasilikelihood information criterion for generalized linear mixed models

https://academic.oup.com/biomet/article/108/2/353/5899715

Phase I

8. Posterior contraction in sparse generalized linear models

https://academic.oup.com/biomet/article/108/2/367/5905471

Phase 0 - pre-clinical!

9. The uniform general signed rank test and its design sensitivity

https://academic.oup.com/biomet/article/108/2/381/5911093

Phase I

10. An assumption-free exact test for fixed-design linear models with exchangeable errors

https://academic.oup.com/biomet/article/108/2/397/5913387

Phase I

11. On quadratic forms in multivariate generalized hyperbolic random vectors

https://academic.oup.com/biomet/article/108/2/413/5897407

Phase 0

12. Estimating differential latent variable graphical models with applications to brain connectivity

https://academic.oup.com/biomet/article/108/2/425/5901536

Phase II

13. Lattice-based designs with quasi-optimal separation distance on all projections

https://academic.oup.com/biomet/article/108/2/443/5866972

Phase II

14. Poisson reduced-rank models with an application to political text data

https://academic.oup.com/biomet/article/108/2/455/5879273

Phase I (?)

15. Finite-time analysis of vector autoregressive models under linear restrictions

https://academic.oup.com/biomet/article/108/2/469/5895298

Phase 0

16. Nonsmooth backfitting for the excess risk additive regression model with two survival time scales

https://academic.oup.com/biomet/article/108/2/491/5869040

Phase II

## Statistical Methods in Medical Research Volume 30(5)

1. Marginal analysis of bivariate mixed responses with measurement error and misclassification

https://doi.org/10.1177/0962280220983587

Phase I-II

2. Harmonizing child mortality data at disparate geographic levels

https://doi.org/10.1177/0962280220988742

Phase II

3. A unified approach to power and sample size determination for log-rank tests under proportional and nonproportional hazards

https://doi.org/10.1177/0962280220988570

Phase III-IV

4. Calibrating validation samples when accounting for measurement error in intervention studies

https://doi.org/10.1177/0962280220988574

Phase I-II

5. A conditional approach for the receiver operating characteristic curve construction to evaluate diagnostic test performance in a family-matched case–control design

https://doi.org/10.1177/0962280221995956

Phase I-II

6. Evaluating Bayesian adaptive randomization procedures with adaptive clip methods for multi-arm trials

https://doi.org/10.1177/0962280221995961

Phase II-III

7. Sample size estimation for modified Poisson analysis of cluster randomized trials with a binary outcome

https://doi.org/10.1177/0962280221990415

Phase II

8. Net benefit separation and the determination curve: A probabilistic framework for cost-effectiveness estimation

https://doi.org/10.1177/0962280221995972

Phase II

9. Quantile regression models for survival data with missing censoring indicators

https://doi.org/10.1177/0962280221995986

Phase II

10. Quantile regression on inactivity time

https://doi.org/10.1177/0962280221995977

Phase II

11. Exposure misclassification in propensity score-based time-to-event data analysis

https://doi.org/10.1177/0962280221998410

Phase I-II

12. A Bayesian dose–response meta-analysis model: A simulations study and application

https://doi.org/10.1177/0962280220982643

Phase II

13. A review of multistate modelling approaches in monitoring disease progression: Bayesian estimation using the Kolmogorov-Chapman forward equations

https://doi.org/10.1177/0962280221997507

Phase III

# Further remarks

Feedback of the raters on the evaluation process was collected via e-mail exchange. This feedback led to the following additional remarks on the classification task:

- Generally, all raters reported that the classification task was difficult, in particular if the methods presented in a paper were outside of the specific expertise of a rater
- The definition of the phases was not yet precise enough to allow for a clear classification, e.g., between phases I and II or between II and III
- Some papers dealt with several methods. For some of the methods, those papers could have been classified as phase III, but then also some new methods were introduced.
- Some non-standard cases were found, e.g., a paper on an efficient implementation of a known method, a paper focusing on a new, more efficient implementation of a known method, and a paper extensively comparing several new methods for a problem without existing method.
- There were even papers which could be classified as a mix of phases I and IV.
- Some papers were not classifiable (because they did not deal with methods) or they presented theoretical work for which a corresponding application was not obvious.

# Conclusion

The authors concluded that a more unambiguous classification would need:

- more precise definitions of the four phases
- a specially designed tool to facilitate the rating
- clear rules how to deal with papers that deal with multiple methods in different phases
- to minimize variation between raters, a paper would have to be assessed by more than one person
